# Deafness the Result of Sympathy between the Ear, Teeth and Mouth

**Published:** 1880-03

**Authors:** Laurence Turnbull

**Affiliations:** Philadelphia


ARTICLE III.

### DEAFNESS THE RESULT OF SYMPATHY BETWEEN THE EAR, TEETH AND MOUTH.

BY LAURENCE TURNBULL, M. D., PHILADELPHIA.

In the author's "Manual of Diseases of the Ear," p. 307, will be found a few hints in reference—

*First*—To the importance of persons suffering with deafness being provided with teeth, as the loss of teeth, causing the soft parts to come together, shuts up the orifices of the Eustachian tubes, which are situated on a level with the turbinated bone, and a little higher than the floor of the nose.

*Again*—The loss of teeth prevents that perfect bony conduction which is so necessary for the vibrations of sound to reach by that avenue the auditory nerve when the other entrance is closed by disease.

I have known, in several instances, when metallic plates were employed for teeth, that an artificial set of teeth would very materially improve the patient's hearing. It is now a well-recognized fact that hard rubber plate will also conduct sound, and that the various forms of the audiphone and dentaphone, with some very deaf persons, has been found useful made of this material.

I have reported a number of instances in my work, where from reflex irritation of the teeth causing deafness, especially in the right and left lower molar, this class of teeth, with the eye and wisdom teeth, are generally the offending agents. In a recent instance, the tenderness and swelling around one of the molars, in the lower jaw, which was plugged, caused pain and tenderness on the floor of the auditory canal, while swelling gradually extended and involved the membrana tympani and side of the face. Not finding the patient willing to have the plug removed, leeches were freely applied at the angle of the jaw, chloroform was used on cotton to the side of the face, and its vapor blown into the ear; also placed around the [illegible] with chloral at bedtime, which had the desired effect of relieving the young lady. A gentleman had a wisdom tooth which for some time gave him pain. Gradually the pain extended to the ear, and was followed by deafness. Various measures were resorted to, to relieve symptoms, by applications to the ear alone, but without any good results. After a considerable time the cause of the trouble was removed by extraction, and in a short time the deafness disappeared. Now, the explanation of this relation of irritation and even inflammation of the ear with pain and deafness, is that the fibrillæ belonging to the vaso-motor system of nerves mingled with those of the cerebro-spinal system; the former set of fibrillæ being brought into reflex relationship with the nervi vasorum distributed on the arteries of the parts reflexly affected by means of the sympathetic ganglia, in which two sets of fibres communicate. In this way distinct channels of communication exist between the vessels and nerves which regulate the supply of blood to the ear and the otic ganglion, while branches of the fifth nerve connected with the carious tooth also communicate with this ganglion. Morbid impressions affecting the latter would influence the former, and thereby produce vascular distension of the membrana tympani and contiguous regions, producing a hyperæmia, followed, if neglected, by pain, inflammation, and, as in the following case, ulceration.

Dr. A——, aged 59, had a very offensive discharge from the auditory canal of the left ear, and below the auricle was an enlarged gland. Various remedies had been tried for the discharge, but no good results followed. Upon examining the ear with a speculum and probe a small ulceration of the bone was found on the floor of the meatus. The teeth were then examined on that side, and one found tender and much decayed. This one was selected as the irritating cause: a molar in the lower jaw of the left side. This tooth was extracted, the bone slightly scraped, and it soon healed, and the discharge from the auditory canal also ceased, and the gland reduced in size.

Explanations of various morbid phenomena of diseased action are here given by this extraordinary reflex action, as, for instance, spasmodic croup, one of the most alarming symptoms of which is a sudden crowing, spasmodic sounding cough from a child at night. The child has had its ear exposed to a draught of cold air, and as a communication exists between the nervi vasorum of the vessels of the larynx and the auriculo-pneumogastro nerve supplying the ear, a reflex action is induced, and you have spasmodic cough. Foreign bodies of various kinds in the meatus also produce a spasmodic cough called aural cough, and persons are suspected of having bronchitis, phthisis. Most of these varieties of cough are relieved by the removal of the offending body. ("A Clinical Manual of the Diseases of the Ear." By L. Turnbull, M. D., 1502 Walnut street. J. B. Lippincott & Co., Philadelphia. pp. 486.)

